# H7N9 influenza split vaccine with SWE oil-in-water adjuvant greatly enhances cross-reactive humoral immunity and protection against severe pneumonia in ferrets

**DOI:** 10.1038/s41541-020-0187-4

**Published:** 2020-05-11

**Authors:** Jørgen de Jonge, Harry van Dijken, Femke de Heij, Sanne Spijkers, Justin Mouthaan, Rineke de Jong, Paul Roholl, Eduardo Alfredo Adami, Milena Apetito Akamatsu, Paulo Lee Ho, Livia Brunner, Nicolas Collin, Martin Friede, José A. Ferreira, Willem Luytjes

**Affiliations:** 1 Centre for Infectious Disease Control, National Institute for Public Health and the Environment (RIVM), Bilthoven, the Netherlands; 2 Central Veterinary Institute of Wageningen UR, Lelystad, the Netherlands; 3 Microscope Consultancy, Weesp, the Netherlands; 4 Influenza Vaccine Plant, Instituto Butantan, São Paulo, Brazil; 5 Seção de Vacinas Aeróbicas, Instituto Butantan, São Paulo, Brazil; 6 Vaccine Formulation Laboratory, University of Lausanne, Epalinges, Switzerland; 7 Initiative for Vaccine Research, World Health Organization, Geneva, Switzerland; 8 Department of Statistics, Informatics and Modelling, National Institute for Public Health and the Environment (RIVM), Bilthoven, The Netherlands; 9 Present Address: Lava Therapeutics B.V., Utrecht, the Netherlands; 10 Present Address: Genmab, Utrecht, the Netherlands

**Keywords:** Adaptive immunity, Infection, Infectious diseases, Vaccines, Infection

## Abstract

Until universal influenza vaccines become available, pandemic preparedness should include developing classical vaccines against potential pandemic influenza subtypes. We here show that addition of SWE adjuvant, a squalene-in-water emulsion, to H7N9 split influenza vaccine clearly enhanced functional antibody responses in ferrets. These were cross-reactive against H7N9 strains from different lineages and newly emerged H7N9 variants. Both vaccine formulations protected in almost all cases against severe pneumonia induced by intratracheal infection of ferrets with H7N9 influenza; however, the SWE adjuvant enhanced protection against virus replication and disease. Correlation analysis and curve fitting showed that both VN- and NI-titers were better predictors for protection than HI-titers. Moreover, we show that novel algorithms can assist in better interpretation of large data sets generated in preclinical studies. Cluster analysis showed that the adjuvanted vaccine results in robust immunity and protection, whereas the response to the non-adjuvanted vaccine is heterogeneous, such that the protection balance may be more easily tipped toward severe disease. Finally, cluster analysis indicated that the dose-sparing capacity of the adjuvant is at least a factor six, which greatly increases vaccine availability in a pandemic situation.

## Introduction

Since the first reported human infections with avian H7N9 influenza virus in China in 2013^1^, the virus has been transmitted from the avian reservoir to humans in a seasonal fashion for several years, although lately the virus appears quiescent^2^. The virus causes a typical influenza-like illness and can progress into a severe viral pneumonia resulting in an acute respiratory distress syndrome (ARDS) accompanied with multiorgan failure^3,4^. Out of the >1500 laboratory confirmed cases, 39% have succumbed to the infection^5^. Epidemiology studies have shown that humans become infected mainly through exposure to virus-infected poultry^6–9^. During the fifth H7N9 infection wave, in 2016/2017, there was a substantial increase in number of human infections and the virus covered a larger geographical area^2,6^. During the fifth wave, several new strains had evolved to become highly pathogenic in poultry (HPAI)^8,10,11^. These isolates had also acquired a multi-basic cleavage site in HA^10,12,13^ and mutations in the PB2 protein^12,14^, which are associated with a wider tissue tropism and pathogenicity^15,16^.

Sustained human-to-human transmission has not been observed, however, mutational analysis has shown that it takes three amino-acid changes to acquire human type receptor specificity^17^. Studies towards evolution of H7N9 viruses in humans on strains isolated from human cases show that these viruses have undergone adaptive evolution and that some sites have evolved in a parallel fashion in different subjects^18^. Some of the amino-acid substitutions are known to contribute to human adaptation. Together, this indicates that H7N9 viruses have a pandemic potential, stressing the importance of preparing for a possible outbreak. The human population is naive to H7N9 in terms of neutralizing immunity, which allows the virus to spread more easily once it becomes transmissible between humans.

The Global Action Plan for influenza vaccines (GAP) of the WHO recommends several strategies to be better prepared for a pandemic^19,20^. One of these strategies is to develop influenza vaccines against potentially pandemic influenza viruses up to clinical phase I/II. As a universal influenza vaccine is not yet available, development of subtype specific vaccines enables rapid response in the event of a pandemic by that specific subtype. Careful consideration of which influenza viruses have a potential to initiate a pandemic is however required and even then, we still may be surprised by an outbreak of an unexpected subtype. Based on pandemic potential of H7N9 influenza, this virus was one of the selected targets for vaccine development. Within the GAP program, we evaluated an H7N9 split vaccine formulated with SWE adjuvant, a squalene-in-water emulsion. With the appearance of new highly pathogenic H7N9 variants and earlier and recent local outbreaks of other H7Nx subtypes^21–23^, cross-reactivity of previously developed vaccines to these variants becomes an important competence within GAP.

Vaccines prepared from H7N9 viruses are considerably less immunogenic in terms of generating neutralizing antibodies than their seasonal counterparts^24,25^. Doses of 30 or 45 µg HA of whole inactivated vaccine virus or 45 µg split vaccine administered twice barely induce haemagglutination inhibition (HI) titers^24,26,27^.

Adjuvants can improve, steer, and broaden the immune response and consequently improve the efficacy of vaccines^28,29^. To overcome the poor immunogenicity of H7N9 vaccines, adjuvants such as aluminum hydroxide, AS03 and MF59 have resulted in a significant improvement of the immune response in humans^24,26,27,30^. In ferrets, addition of adjuvants greatly improved protection^31,32^ even against antigenically distinct HPAI H7N9 infection^33^. However, a major obstacle for application in pandemic influenza vaccines in low-income countries is the potential lack of access to proprietary adjuvants.

Aluminum hydroxide is a commercially available adjuvant but only induces a modest pandemic influenza antigen-sparing capacity compared with oil-in-water emulsions such as the proprietary adjuvants AS03 (GlaxoSmithKline) and MF59 (Novartis)^24,27^. We therefore selected the SWE adjuvant, which is free of intellectual property rights. This adjuvant has been developed by the Vaccine Formulation Laboratory for technology transfer in order to empower developing countries vaccine manufacturers to increase pandemic influenza vaccine capacity^34,35^. SWE is an oil-in-water emulsion of similar composition to MF59, and has previously been successfully used in preclinical studies with various vaccine candidates against poliovirus^36^, Leishmania^37^, RSV^38^, Malaria, Hepatitis B, Tuberculosis^39^, and rabies^40^ and is awaiting clinical testing.

Here, we evaluate an H7N9 split vaccine formulated with or without SWE adjuvant in the ferret model at different dose levels. We show that addition of the adjuvant has a strong immunopotentiating- and therefore dose-sparing effect and allows for the induction of cross-reactive antibodies against H7 strains from different lineages. Both vaccines protect against severe pneumonia induced by intratracheal infection, whereas the adjuvanted vaccine requires a lower dose of antigen to accomplish this. Furthermore, cluster analysis indicates that ferrets vaccinated with the non-adjuvanted vaccine may be more easily pushed towards severe disease progression.

## Results

### Antibody response

**Fig. 1:**
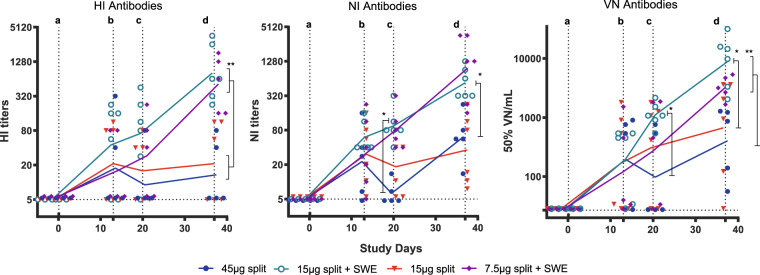
Functional and neutralizing antibody responses. Haemagglutination inhibition, neuraminidase inhibition, and virus neutralization antibody titers detected during the course of the study in serum of ferrets that received either the split vaccines alone (45 µg HA—blue circles, 15 µg HA—red triangles) or the SWE-adjuvanted split vaccines (15 µg HA—green open circles, 7.5 µg HA—purple diamonds). HI-Titers were measured against four haemagglutinating units H7N9 (A/Anhui/1/2013) using a concentration of 1% horse erythrocytes. NI-titers were determined using an H6N9 (RN19/13-human reassortant strain) dilution with a neuraminidase activity resulting in an ~OD 1 after 1 hour incubation at 37 °C. Inhibition of neuraminidase was considered at an OD of less than three times standard deviation below the maximal OD. VN-titers were measured against 100 TCID_50_ of H7N9 and 50% Virus neutralization was calculated according to Reed and Muench^70^. Presented are the individual titers and a line connecting the geometric means. *p < 0.05; **p < 0.01, by the WMW test. Horizontal dotted line: detection limit. Vertical dotted lines indicate: a. 1st vaccination; b. blood collection; c. 2nd vaccination; d. challenge.

When ferrets were immunized with split vaccine without adjuvant, low antibody levels were detected in all functional assays irrespective of dose, which in general declined after day 13 (Fig. 1). In half of the animals, no HI-titer was detected and a second vaccination 21 days later did not boost the HI-response. Booster vaccination did increase NI- and VN-titers, and these were detectable by day 37 in all split-vaccinated animals (*P* < 0.01 vs. placebo).

Addition of the SWE adjuvant had a clear immune-potentiating effect, as the titers of the functional antibodies were significantly higher than in the placebo and split alone vaccine groups (Fig. 1). The majority of the animals responded after a single administration (*P* < 0.05 vs placebo for VN- and NI-titers at day 13 and 20). After booster, high titers in all animals in all assays were detected (*P* < 0.01 vs placebo). The HI and VN responses at day 37 were significantly higher than in the two split vaccine without adjuvant groups. The NI-titers were significantly higher for the 15 µg adjuvanted vaccine compared with the 45 µg split alone vaccine at day 20 and 37. There was no measurable difference between the groups immunized with different doses of the adjuvanted split vaccine in either of the assays.

### Cross-reactive antibody response

**Fig. 2:**
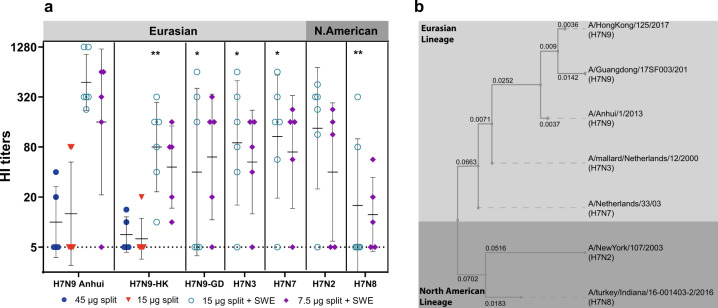
Cross-reactive antibody responses. Cross-reactive antibody responses were determined by haemagglutination inhibition **a** Groups that did not show any cross-reaction were left out. Presented are the individual titers, geometric means, and SDs. Horizontal dotted line: detection limit. Significant lower responses compared to the homologous strains are indicated by *(*p* < 0.05) and **(*p* < 0.01), by the WMW test. split vaccines alone: 45 µg HA—blue circles, 15 µg HA—red triangles, or the SWE-adjuvanted split vaccines: 15 µg HA—green open circles, 7.5 µg HA—purple diamonds. A phylogenetic tree of the divergent strains tested for cross-reactivity is shown in **b**. H7 HA’s are divided in two lineages, the Eurasian and North American lineage. The numbers indicate branch length.

To investigate potential cross-protection against divergent H7 strains, we assessed cross-reaction by HA-inhibition against H7 strains from the Eurasian and North American lineages (Fig. 2), including two Eurasian highly pathogenic H7N9 strains that recently emerged and were selected as new H7N9 vaccine candidates by the WHO^41^.

Homologous HI-titers generated by the split vaccine alone were mostly absent, accordingly cross-reaction was not detected, except for some responses against the H7N9-HongKong strain. In contrast, the adjuvanted vaccines showed strong cross-reaction against all tested strains with exception of the H7N8 strain. In general, the average cross-reactive HI-titers were ~2–3 dilutions lower than the titers against the homologous strain and ranged between 40 and 160. Even the response against the H7N2 virus, which is from a different lineage (North American) was within this range. Cross-reactivity against the H7N8 strain of the North American lineage was clearly lower than against the other strains and showed more non-responders.

### Clinical manifestations

**Fig. 3:**
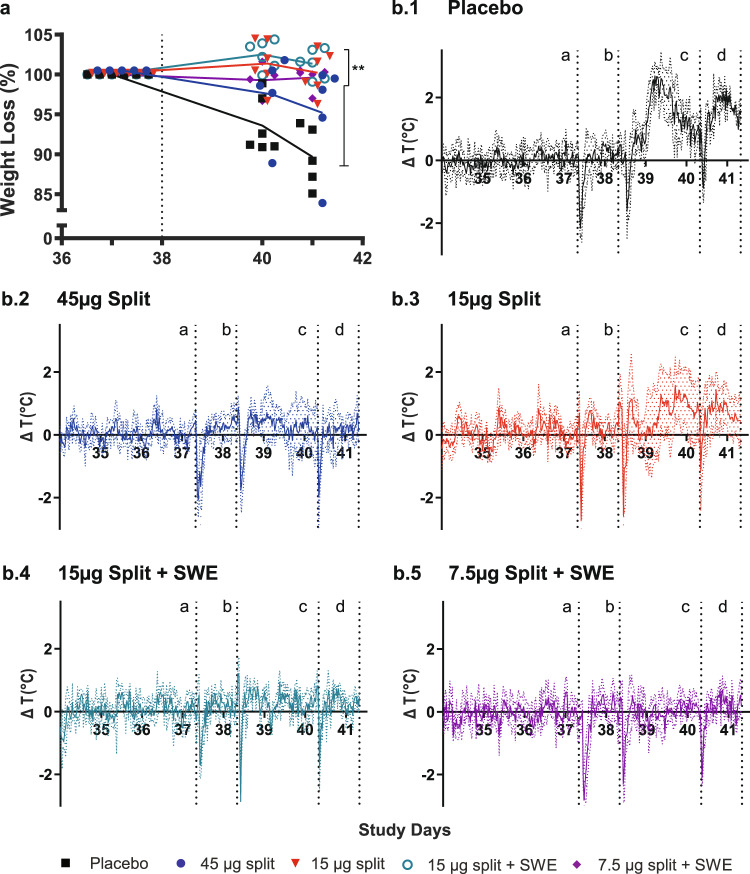
Weight loss and fever. Body weight prior to challenge was set at 100% and percentage weight loss was subtracted from baseline **a**. Presented are the individual data and lines connecting the averages: placebo (black squares), split vaccines alone (45 µg HA—blue circles, 15 µg HA—red triangles) or SWE-adjuvanted split vaccines (15 µg HA—green open circles, 7.5 µg HA—purple diamonds). Body temperature was recorded every 30 min by transponders that were placed in the intraperitoneal cavity. To analyze fever development, the temperature difference compared with baseline was calculated and the average ΔT per group was plotted. The colored dotted lines indicate the standard deviation. The several sharp decreases in temperature are a result of sedation required for sampling and challenge. Color coding is the same as for the body weight graph. b.1 placebo; b.2 45 µg split; b.3 15 µg split; b.4 15 µg split-SWE; b5 7.5 µg split-SWE. Vertical dotted lines indicate: a. challenge; b and c. swab collection; d. termination. WMW test: **p < 0.01.

When the placebo-vaccinated ferrets were challenged via the intratracheal route 17 days after booster immunization, they became inactive and developed ARDS 2–3 days post challenge (d.p.c., supplementary Table 1). A strong fever peak developed within 24 h (max. ∆T of ~3 °C) followed by a second peak 2 d.p.c. (Fig. 3). The average temperature increase during the interval after challenge was ~1.2 °C (supplementary Table 2). By 2 d.p.c., the ferrets had lost ~5% weight and at termination (3 d.p.c.) weight loss had increased to ~10% (Fig. 3, supplementary Table 2). Finally, two animals died and one animal had to be killed prior to scheduled termination.

None of the vaccinated animals had to be killed prior to scheduled termination and except for one ferret, none showed the typical clinical symptoms that accompany an intratracheal H7N9 infection (supplementary Table 1). All vaccines, except for the 45 µg split vaccine alone prevented weight loss in all animals (Fig. 3, supplementary Table S). In the 45 µg split vaccine group reduced weight loss was observed as the maximum weight loss was less compared with the placebo group (supplementary Table S). Only the adjuvanted vaccines and the 45 µg split vaccine alone prevented the development of fever. With the 15 µg split vaccine alone, fever was still observed: the maximum temperature increase was significantly higher than in the other three vaccine groups and the average temperature increase significantly higher than in the 7.5 µg adjuvanted group (supplementary Table 2). One ferret in the 45 µg unadjuvanted split vaccine group (ID #07) developed severe disease similar to the placebo group with less activity, heavy breathing and substantial weight loss; however without the typical fever.

### Virus replication in the respiratory tract

**Fig. 4:**
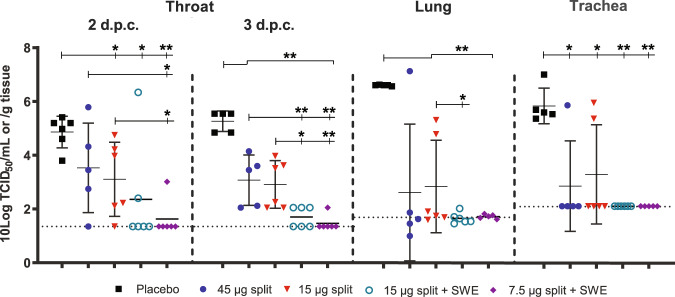
Virus replication in the respiratory tract. Virus titers in the throat, 2 and 3 days post challenge (d.p.c.) and in the trachea and lung 3 d.p.c. in ferrets vaccinated with placebo (black squares), split vaccines alone (45 µg HA—blue circles, 15 µg HA—red triangles) or SWE-adjuvanted split vaccines (15 µg HA—green open circles, 7.5 µg HA—purple diamonds). The virus titers in the transport buffer of the swabs or homogenized tissue samples were determined by end-point titration on MDCK cells using a fivefold serial dilution. Presented are the individual 10 log-transformed titers, their averages (bar) and SD (error bar). Dotted horizontal lines indicate limit of detection. One animal of the placebo group was excluded as this animal died ~1 day before section. Another animal of the placebo group that died prior to section was still included as the time of death was only a few hours before section. WMW test: *p < 0.05; **p < 0.01.

In placebo-vaccinated animals, high virus titers of up to ~5.5 logs (TCID_50_) were detected in the throat two and three d.p.c. and in the trachea (~5.5 logs) and lung (~6.5 logs) three d.p.c. (Fig. 4). Vaccination with 45 and 15 µg split vaccine alone reduced virus replication in the throat significantly at 3 d.p.c., but virus titers were still detectable. Viable virus in trachea and lung could not be detected, except in three ferrets (ID#07, #12, and #13) with high virus titers. Vaccination with adjuvanted split vaccine reduced virus replication compared to the split vaccine alone, as throat swabs taken two and three d.p.c. contained significantly lower virus titers. Moreover, virus could not be detected in the trachea and lungs of ferrets immunized with adjuvanted split vaccine.

### Lung pathology evaluation

**Fig. 5:**
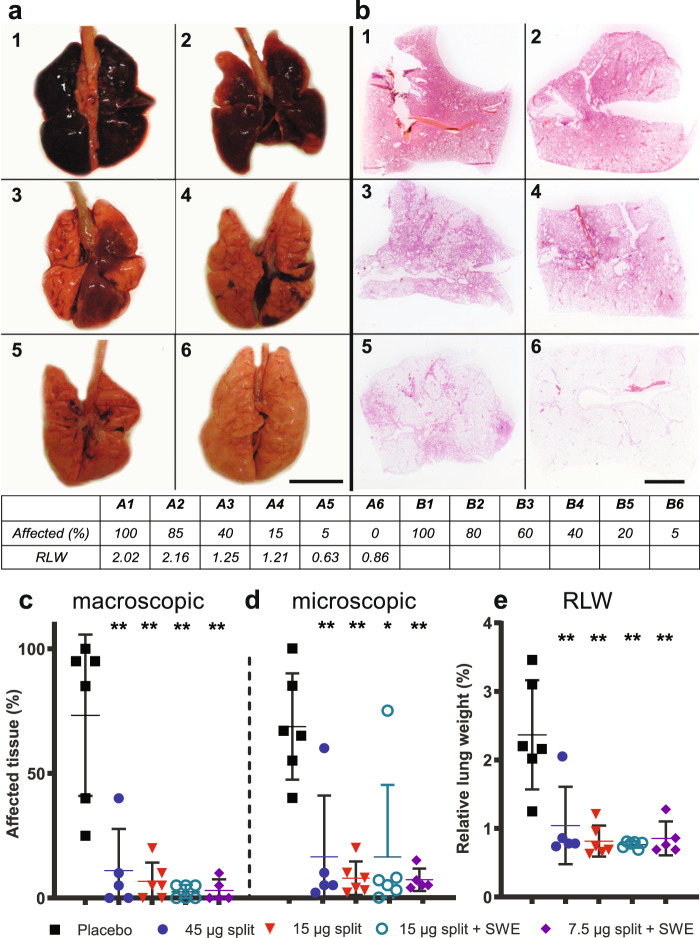
Macroscopic and microscopic illustrations and analysis of lung pathology. Macroscopic illustrations of the lungs **a** and microscopic illustrations of hematoxylin and eosin HE-stained lung sections **b** to exemplify the scoring of the percentage affected lung tissue (shown in the table). **a** and **b**, numbers 1–4 are typical for the placebo group and numbers 5 and 6 for the vaccine groups, excluding the outliers. Means and SDs of macroscopically **c** and microscopically **d** estimated percentages of affected tissue. The relative lung weight (RLW) was calculated as the ratio of the lung weight at termination to body weight at the day of challenge **e**. Corresponding RLW’s to the illustrations in **a**, are shown in the table. Bar in **a**: 3 cm and bar in **b**: 0.5 cm. WMW test: *p < 0.05; **p < 0.01 compared with the placebo group.

Lungs of placebo-treated ferrets appeared opalescent, edematous, and swollen, three d.p.c. with H7N9 virus. In four out of six ferrets, >80% of the lung surface contained dark lesions (Fig. 5a, c) and microscopically 40–100% of the lung parenchyma was affected (Figs. 5b, d). The infection had less impact on two ferrets of which only <50% of the lung surface was affected. Owing to edema accumulation, lungs weighed 3–5 times more than lungs of healthy ferrets and ferrets showed a higher relative lung weight (RLW, Fig. 5e) which is an indicator for advanced pneumonia. The ferret of which 25% of the lung was affected showed a clearly increased RLW of 2.2, indicating initiation of severe pneumonia.

In animals vaccinated with H7N9 split vaccine, either with or without adjuvant, lungs appeared normal with <10% of the surface affected (Fig. 5a, c). Less than 10% of microscopically examined lung parenchyma (Figs. 5b, d) was affected and the RLW was at or close to baseline (Fig. 5e). However, ferret #07 (45 µg split vaccine) had clearly affected lungs (40%) and a high RLW (2.05) and one ferret (#17) from the 15 µg split adjuvanted vaccine group had increased affected lung tissue (microscopically). These effects were only visible macroscopically as a scattering of small spots. There were no significant differences between the different dosages or between the adjuvanted and non-adjuvanted vaccines.

### Histopathological evaluation of lung pathology

**Fig. 6:**
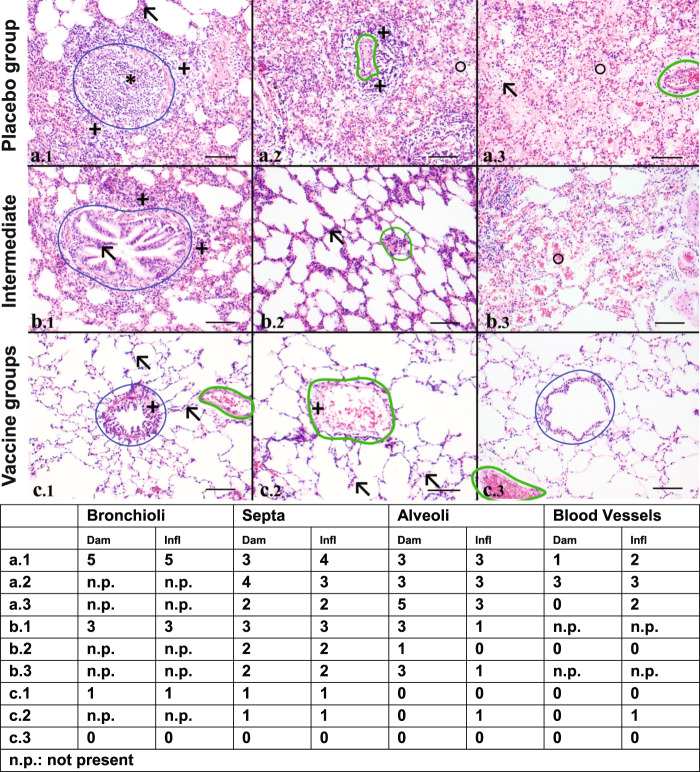
Detailed histopathological analysis of the lung. **a**–**c** Representative hematoxylin and eosin (HE)-stained lung sections to illustrate the scoring system of pathology parameters. Scores of the pathology parameters that go along with the images (a.1-c3) are indicated in the table. All individual scores are presented in supplementary Fig. 1. Bronchioli and blood vessels are marked in blue and green, respectively. Enlargements: ×10 objective, bar represents 100 µm. **a** images a.1–3 are representative sections for the placebo groups and illustrate necrotizing denudation of bronchioli with luminal plugs of inflammatory exudate (a.1,*), accompanied by a large peribronchiolar rim of lymphocytes and some polymorphonuclear (PMN) cells (a.1,**+**). Inflammation is spread into the interstitium (a.1, **↖**) and alveolar space (a.3, **↖**). Hyperemia is strongly present and alveolar space is filled with edema, fibrin, and debris and PMN are regularly present (a.2 and A.3, **○**). Inflamed small arteries with perivascular inflammation are also observed (a.2, **+**). **b** images b.1–3 are typical for a few placebo- and split-vaccinated animals that were either less or more affected than the general trend, respectively. Bronchioli with moderate peribronchial inflammation (b1, **+**) and hyperplasia of epithelium (b.1, **↖**). Alveolar septa with mild or slight (b2, **↖**) inflammation and hyperemia and alveoli filled with erythrocytes (b3, **○**). **c** images c.1–3 are representative for the split-vaccinated ferrets. Observable are a bronchiolus with minimal peribronchiolar inflammation and hyperplasia of epithelium (c.1, **+**), alveolar septa with minimal inflammation and hyperemia (c.1, c2 **↖**) and minimal inflammation of the alveoli (c.2). The small vene minimally inflamed (c.2, **+**). c3 shows a normal histology of the lung parenchyma. Presence of normal bronchiolar epithelia and a blood vessel, regular alveoli with thin septa and absence of enhanced cellularity.

Figure 6 shows representative histopathological illustrations of lungs from placebo and vaccinated ferrets and the scores for the different parameters per compartment to illustrate the scoring system. A full analysis for the individual ferrets is presented in supplementary Fig. 1.

Intratracheal infection with H7N9-induced severe pathology in five out of six animals of the placebo group, characterized by a moderate to strong bronchopneumonia, which spreads into the interstitium and alveoli (Fig. 6a). Bronchiolar lumens are regularly obstructed by exudates, with necrosis of bronchiolar epithelium of inflamed bronchiolar walls (a1). Furthermore, inflammation was also substantial in interstitial septa and in the alveoli (a3). In addition, there was substantial alveolar damage characterized by influx of edema, fibrin and debris, inflammatory cells, and sometimes hemorrhage into alveolar space (a2 and a3). A mild or strong inflammation is observed in part of the blood vessels (a2).

In most vaccinated animals (Fig. 6c) the infection pathology was restricted to a minimal peribronchiolitis (c1), sometimes combined with a minimal perivasculitis and inflammation of the interstitium (c2). Inflammation in the alveoli was absent. Damage of the epithelial cell lining in the bronchioli was minimal (c1), absent to minimal in the interstitium (c1–3) and in most cases absent in the alveoli (c1). Panel b represents intermediate examples of a few animals that were either from the placebo group and showed milder effects than the group mean or from the vaccinated ferrets that showed more pronounced effects than the general trend (See supplementary Fig. 1).

**Fig. 7:**
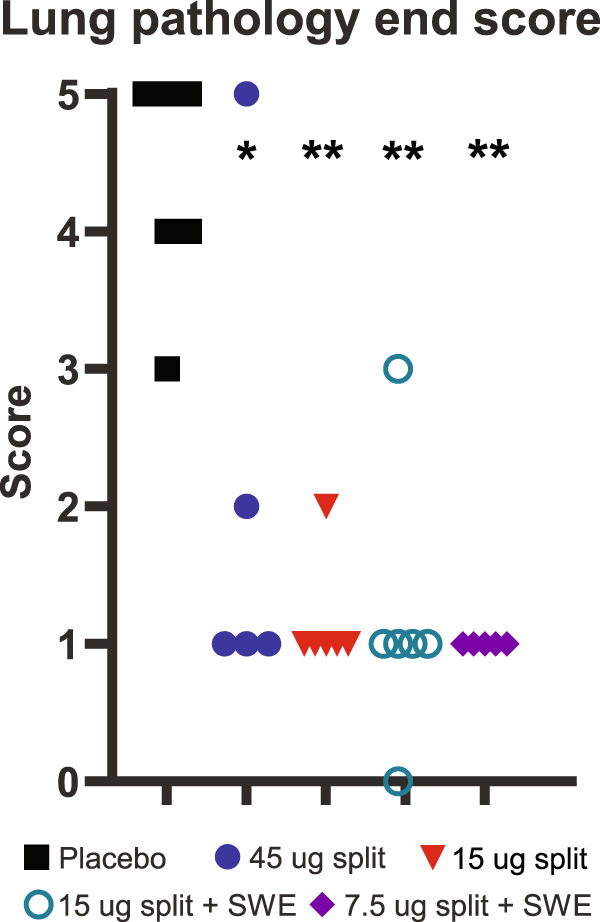
Pathology ‘end score’. The end score is an overall pathology score, which is determined based on the amount of tissue affected, the degree of inflammation and the amount of damage in the left caudal and cranial lung lobe. The scores range from 0 (no aberrations) to 5 (severely affected). Statistical analysis was performed using an adapted version of the Mann–Whitney test for ordinal data with a small sample size: *p < 0.05; **p < 0.01 compared with the placebo group.

An end score of the overall infection pathology for the lung was determined, taking into account the amount of tissue affected, the degree of inflammation and the amount of damage (Fig. 7). The end scores for placebo-vaccinated animals ranged from 3 to 5. In general, vaccination reduced these scores to 1 in all the different vaccine-treated groups. Ferret #07 from the 45 µg split group also developed a full-blown lung pathology (score 5) and two ferrets from the non-adjuvanted vaccine groups showed minimal pathology (score 2). In addition, a ferret in the 15 µg split adjuvanted vaccine group showed a moderate pathology (score 3). The latter was not reflected in the clinical symptoms, virus replication and macroscopic lung pathology of this animal. Between the vaccine groups there were no significant differences with regard to lung pathology.

### Correlation between antibody response and efficacy parameters

**Fig. 8:**
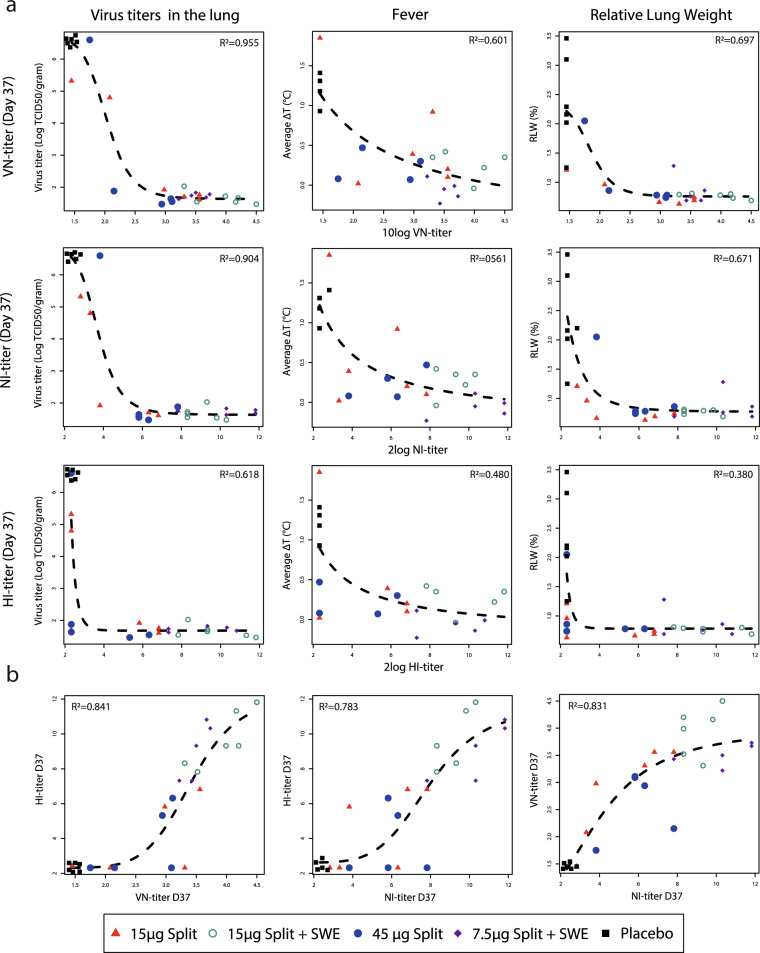
Correlation analysis between functional antibody responses and vaccine efficacy parameters. **a** Correlation between VN-, NI-, and HI-titer and virus replication in the lung, fever, and pathology visualized by interpolation (dashed line) using the sigmoid emax model for virus titers and RLW and the Emax model for fever. Antibody titers were determined at the day of challenge (day 37). Vaccine treatment is indicated by shape and color. *R*^2^ indicates the goodness of the fit. Lung viral titers were selected because the lung is the organ of direct vital importance and should be protected. Fever and relative lung weight (lung pathology) as clinical and pathological parameters were preferred, because they contained more measurements and/or are not based on a subjective scoring system. **b** Correlation between the different antibody titers at day of challenge visualized by interpolation using the sigmoid Emax model.

To be able to link vaccine efficacy to immunological parameters, we studied the correlation between functional antibody responses and vaccine efficacy parameters. We found strong associations between these parameters using the independent test, most associations had a *p* value < 0.0005 (Supplementary Table 3). Next, representative read-outs for virus replication, clinical disease and pathology were selected and curves were fitted to the data to reveal the nature and accuracy of the correlations (Fig. 8a).

The curves best describe the correlation between NI- and VN-titers and lung viral titers (*R*^2^ > 0.90; Fig. 8a). There is also a good correlation between NI- and VN-titers and RLW (*R*^2^ > 0.67), but less between antibody titers and fever (*R*^2^ ≤ 0.60). Strikingly, the correlation between the HI-titers and efficacy parameters was poor, as *R*^2^ was much lower than for the NI-and VN-titers. This reflects the, in general, higher *p* values for associations between the HI-titer and efficacy parameters in the independent test (supplementary Table 3).

Based on the fitted curves, the level and nature of protection provided by antibody titers was estimated. When vaccines induce VN-titers of ≥400 and NI-titers of ≥40, virus replication in the lung remains below detection level. To protect against lung pathology, lower antibody levels are required: ≥160 VN-titers and ≥20 NI-titers, determined at 1% RLW (baseline). The curves cannot be used to determine protective levels of HI-titers owing to the great uncertainty within the lower range of the observations. However, at HI-titers of ≥40, no virus replication and lung pathology are observed. Severe fever is already reduced at titers below above mentioned levels. On the other hand, fever cannot be totally prevented and, despite vaccination, a mild fever is still likely to occur.

The poorer sensitivity of the HI-assay is further illustrated in Fig. 8b, which shows that VN- and NI-titers are already detected when no HI-response is observed. Contrary, VN- and NI-titers show an initial linear relation, implying equal sensitivity. The sigmoid Emax model (highest *R*^2^ of the four tested models) fitted to the data underline these observations.

### Cluster analysis

To analyze and comprehend large data sets, algorithms have been developed to visualize associations between objects. Cluster analysis is a treatment-blind descriptive algorithm that identifies groups (clusters) of similar objects within a population. We used this analysis to visualize general patterns in the ferret study population with respect to vaccination and how individual animals relate to these patterns.

As a representative for the overall immune potency of the vaccines, including the effect of priming and boosting, the area under curve (AUC) of the log-transformed antibody titers in time was calculated for each functional antibody assays and used for cluster analysis of the antibody response. This clusters ferrets based on their similarity in immune responses. To visualize the overall vaccine efficacy, a second cluster analysis was performed on selected efficacy read-outs, including all virus replication, weight, fever and pathology (excluding ordinal data) parameters. Both data sets passed the Hopkins test (*H* value <0.5), meaning they were clusterable (*H* value: 0.29 and 0.25 for antibody response and efficacy, respectively).

As part of cluster analysis, distances between all combinations of two ferrets were calculated with the Euclidean method for both data sets. These are represented in dissimilarity matrices that show the distance between two objects (supplementary Fig. 2A). The distance calculation was used as input for the partitioning around medoids (PAM) clustering algorithm to identify clusters. Next, we determined the number of clusters by performing the analysis using 2–5 clusters followed by a cluster validation test using a silhouette plot (supplementary Fig. 2B). This approach showed that, either two or four clusters for both data sets were optimal. In our analysis, we proceeded with four clusters as this revealed more detail.

**Fig. 9:**
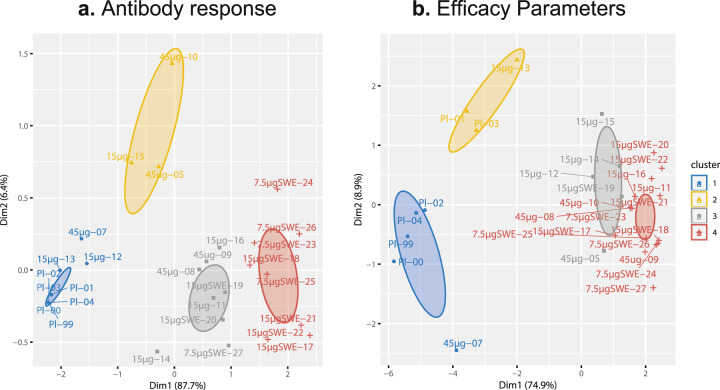
Cluster analysis of the antibody response and efficacy measurements. Cluster analysis using the partitioning around medoids (PAM) algorithm as a descriptive statistical approach to visualizer overall trends in the ferret study population. Subjects are plotted using a principal component analysis and clusters determined by PAM are indicated by ellipses. **a** Clustering of the antibody response based on the area under curve (AUC) of the log-transformed antibody titers from D0 until D37. **b** Clustering of the efficacy parameters based on assays with numerical data output including virus replication, weight, fever, and pathology parameters (excluding ordinal data). Labels indicate vaccine dose with or without adjuvant and animal number. *Pl* placebo.

For the antibody response, Fig. 9a shows the four clusters identified in the ferret study population that range from no or low antibody responses (blue#1 and yellow#2 cluster) to medium responses (gray#3 cluster) to high responses (red#4 cluster). The adjuvant effect is clearly visible as cluster 4 only contains the adjuvanted vaccine and only three animals of the adjuvanted groups are located in cluster 3. The non-adjuvanted vaccinated ferrets respond much more heterogeneous as these are located in three clusters that represent medium to no antibody response. The latter includes all the placebo animals.

The four clusters that are identified within the efficacy data set decrease in severity from left to right (Fig. 9b). They can roughly be divided into two categories that either include animals with severe disease (blue#1 and yellow#2 cluster) or those that include animals that are protected (gray#3 and red#4 cluster). The yellow cluster (#2) contains less severely affected animals compared with the blue cluster (#1). Subjects in the gray cluster (#3) show more mild disease/pathology or virus replication than ferrets in the red cluster (#4), who are almost completely protected.

Addition of the SWE adjuvant evidently improves the efficacy of the vaccine, as all animals except one that received this vaccine are located in cluster 4 (Fig. 9b). Again, the non-adjuvanted vaccine leads to a heterogeneous distribution over the clusters as some ferrets (*n* = 5) are in cluster#4, whereas others (*n* = 4) are in cluster#3 and two ferrets are in cluster #1 or #2. A dose effect could not be confirmed, as both non-adjuvanted groups are located in the different clusters. Likewise, a dose effect for the adjuvanted vaccine could not be established, as ferrets locate to just one cluster.

Animals that belong to the highest antibody response cluster (#4 in Fig. 9a), also belong to the cluster that represent near complete protection (cluster#4, Fig. 9b). Likewise, ferrets in cluster 1 (Fig. 9a) localize to clusters 1 or 2 in Fig. 9b, representing severe disease, although there are exceptions, as animal #12 localizes to the gray cluster (#3, Fig. 9b) in the efficacy analysis. The connection between the clusters of the two analysis shows there is a relation between the antibody titer and protection, but that a low antibody response not necessarily predicts disease. These observations are consistent with the correlation analysis performed above.

Thus, cluster analysis provides a representative overview of all the results and reveals individual ferrets that respond differently than the group trend. The analyses show that addition of the adjuvant results in a higher immune response and a far more robust efficacy and that a low dose (7.5 µg HA) is sufficient. When immunizing with the split alone vaccine, the animals may much easier tip towards severe disease progression, as many of the animals are in a cluster closer to the placebo group and some ferrets already localize with the placebo group clusters.

## Discussion

We here examined an H7N9 split vaccine, either with or without the SWE oil-in-water adjuvant, at different dose levels and found that the adjuvant clearly enhanced haemagglutinin- and neuraminidase inhibition and virus-neutralizing antibody responses. The adjuvanted vaccine protected against severe pneumonia and virus replication and clinical signs, such as weight loss and fever were almost absent. Despite an impaired induction of a humoral response, the non-adjuvanted split vaccine protected in almost all cases from severe pneumonia, but virus replication, fever, and weight loss were less successfully prevented. Using cluster analysis, we visualized that the addition of the adjuvant results in robust protection and that a low dose was sufficient, whereas the protection induced by the split-alone vaccine is heterogeneous, indicating that in these animals the balance may be more easily tipped toward severe disease progression.

Potentially pandemic H7N9 influenza virus is low immunogenic in humans^42^ and consequently even high doses (30–45 µg HA) of whole inactivated virus vaccines or split vaccines induce inconsistent and low HI-titers in humans^24,26,27^ and in ferrets^31,43^. The latter underpins that the ferret model reflects the human situation and adds to the ability to extrapolate ferret antibody results to the clinic. In our study, these observations were confirmed as split vaccine alone (15 and 45 µg HA) induced an antibody response only in half of the animals, which declined rapidly and boosting did not increase HI or NI titers. Addition of adjuvants can overcome the low immunogenicity of H7N9 as several preclinical and clinical studies have shown^24,26,27,31^. We here show that the SWE adjuvant effectively boosts functional antibody responses (VN-, NI-, and HI-titers).

The usability of the vaccines tested during GAP stands or falls with their cross-reactive potency, as H7N9 diverges^2,6^. We therefore investigated cross-reactive HI-titers against strains from the Eurasian as well as the North American lineage and recently emerged HPAI H7N9 strains. Only the SWE-adjuvanted vaccines induced cross-reactive HI-antibodies of significant titers against Eurasian strains, including the newly emerged HPAI strains and against an H7 strain of the North American lineage. Previously Hatta et al.^33^ showed that a LPAI H7N9 whole-inactivated virus vaccine cross-reacted and provided protection against HPAI H7N9 challenge in ferrets. Also others have shown that H7 vaccines cross-react and protect against drifted H7 influenza strains in mice^44^. Therefore, the cross-reactive HI-titers found in this study are an indication that the SWE-adjuvanted split vaccines may cross-protect against divergent H7 strains.

Ferrets are readily infected by influenza strains that infect humans and express comparable clinical manifestations^45–47^, partly owing to the similar lung physiology and sialic acid receptor distribution in the respiratory tract. Often the intranasal infection model is used for investigating vaccine effectiveness^31,32,43,48,49^, however for H7N9, this route of inoculation results in fairly mild disease^50–53^, which does not reflect the disease observed in the severe human cases^1,3,4,54–56^. Also, lung viral titers could not be reproducibly induced through this route^31,52^. Results from these studies are therefore difficult to extrapolate to humans in terms of protection against disease. They should be viewed in the light of prevention of virus replication in the upper respiratory tract, which is an obvious important parameter in a pandemic situation as this would limit transmission. Despite the unnatural route of infection, intratracheal inoculation of ferrets with H7N9 induces severe bronchopneumonia resulting in ARDS^53,57^, in line with clinical findings in severe cases^1,3,4,54–56^. Vaccine studies in this model^53,58^ likely have a better predictive value for effectiveness against disease in humans. We here show, by detailed analysis of the clinical manifestations and in depth pathological analysis, that the adjuvanted split vaccine and, to a lesser extent, the unadjuvanted split vaccine protect against severe pneumonia and ARDS induced by intratracheal infection. The limitation of the i.t. model is that there is no consistent virus replication in the upper respiratory tract, and therefore well-founded conclusions with respect to shedding cannot be made. Yet, it may be assumed based on strongly limited virus replication in the throat and lung, that the split vaccines would limit virus shedding too. Therefore, the route of infection should be carefully considered with regard to which protection parameter is investigated to determine vaccine efficacy.

As the split vaccines induced a range of functional antibody titers, the correlation of these responses with protection against infection could be studied. There was a strong association between functional antibody responses and vaccine efficacy parameters. Higher values of *R*^2^ are observed in the relations involving VN- and NI-titers than in those involving HI-titers. Curve fitting showed that protection increased sigmoidally with increasing NI- and VN-titers, whereas for HI-titers only when above a threshold there was a clearer correlation with protection. It was calculated that VN-titers of ≥400 and ≥160 and NI-titers of ≥40 and ≥20 protected against virus replication in the lung and lung pathology, respectively. However, these are indications as for accurate estimates more data are required. Animals were protected from severe fever at low functional antibody titers, but mild fever could not be completely prevented, even with high functional antibody titers. In addition, correlation analysis showed that the NI-, and VN-data are more predictive in the lower range than the HI-data and when NI-, and VN-titers could not be detected, the animals were not protected. In contrast, when HI-titers were absent, some animals were still fully protected. Therefore, the NI and VN antibody titers are each independent predictors for vaccine efficacy and predictions based on the HI-assay may lead to an underestimate of protection. This has been observed in other studies in ferrets^43^ but also in humans^59^.

NI-antibody titers have been shown as an important correlate of protection^59–63^. Recently, the waning vaccine effectiveness for H1N1 has been attributed to immune escape of NA, whereas HA did not show significant antigenic drift^64^. In two human challenge studies NI was the only independent predictor for clinical outcome^59,62^. The results of the current study are in line with the findings in the clinic and stress that Neuraminidase should be included in sufficient amounts in the vaccine to assure a better and broader efficacy.

We used cluster analysis, which is an unbiased algorithm that identifies groups of subjects in a population, called clusters, based on similarity, to analyze the disparate data set in this study. We analyzed the antibody response results and the efficacy results separately. The outcome showed that ferrets immunized with SWE-adjuvanted vaccine localized to the cluster with the highest antibody response and to the cluster containing ferrets that were best protected against challenge. The efficacy was very robust as only one animal fell out of the latter cluster. Vaccine dose differences had no effect on clustering. For the non-adjuvanted vaccinated animals, clustering was highly heterogeneous both for the antibody responses and efficacy measurements. Animals located in several clusters, including those that contained placebo animals. The added value of cluster analysis is that it provides a clearer picture of the performance of the different vaccine formulations than when considering assays individually. It also helps in interpreting possible outliers or a high variation in assay outcome and shows that these may be indicative of a fragile protection balance that can easily be pushed towards severe disease in non-adjuvanted split-vaccinated ferrets.

Vaccine production capacity is limited and not sufficient in the event of a pandemic. A substantial dose-sparing capacity of an adjuvant is therefore an important requirement. The cluster analyses show that the SWE-adjuvanted low dose (7.5 µg) vaccine performs better than the high dose (45 µg) split alone vaccine with respect to the antibody response and vaccine efficacy. Thus, addition of the SWE adjuvant to the split vaccine increases the amount of vaccines that can be produced by at least a factor six, which is crucial for vaccine availably in a pandemic situation.

Other vaccines against H7N9 with a previously proven seasonal track-record have been developed. These have shown to be effective in preclinical models and immunogenic in the clinic and include live attenuated influenza vaccines^53,65^, and split and whole-inactivated virus vaccines^24,26,27,30^. Each vaccine will have its pros and cons, but in the face of a pandemic all these vaccines will be required, as the major limitation will be the production capacity^66^. Here, we show that adjuvants can play an important role, not only in the efficacy of the vaccine, but more importantly in the dose-reduction effect and cross-reactive response induction. Until universal influenza vaccines becomes available with lasting efficacy, developing and testing “classical” vaccines up to clinical phase I/II studies and including adjuvants to broaden the immune response remains the road to travel and the current study contributes to this strategy.

## Methods

### Ethical statement

The animal experiments were ethically approved by the Committee on Animal Experimentation of the Antonie van Leeuwenhoek terrain (DEC-Alt, Bilthoven, the Netherlands). Animal handling was carried out in compliance with relevant Dutch national ethical legislation, including the 1997 Dutch Act on Animal Experimentation. Animals were examined for general health on a daily basis. After challenge, ferrets were scored twice daily for activity, impaired breathing, and survival. The following scoring system was used for activity: 0=active; 1=active when stimulated; 2=inactive and 3=lethargic; and for Respiratory distress: 0=normal breathing; 1=fast breathing and 2=heavy and stomach breathing. If animals showed severe disease according to the defined end points (lethargic or heavy breathing and inactive or more than 20% weight loss) prior to scheduled termination, they were killed by cardiac bleeding under anesthesia with ketamine (5 mg/kg) and medetomidine (0.1 mg/kg).

### Viruses

H7N9 A/Anhui/1/2013 wild-type influenza virus as well as H7N9 A/Guangdong/17SF003/2016 reassortant (NIBRG-375) and H7N2 A/NewYork/107/2003 reassortant (NIBRG-109), both prepared by reverse genetics, were obtained from the NIBSC under the conditions of the Pandemic Influenza Preparedness (PIP) framework. A/Netherlands/33/03 (H7N7) and A/mallard/Netherlands/12/00 (H7N3) were obtained from the repository of the Centre for Infectious Disease Control of the RIVM (Bilthoven, the Netherlands). The H7N9 vaccine strain (A/Shangai/2/2013(H7N9)-PR8-IDCDC-RG32A.3) and the wild-type H7N9 A/HongKong/125/2017 were provided by the CDC (Center of Disease Control, USA) and H7N8 A/Turkey/Indiana/16-001403/2016 was obtained from the Animal and Plant Health Inspection Service from the USDA. RN9/13-human A(H6N9) reassortant was obtained from the Institute of Experimental Medicine (St. Petersburg, Russia)^53^.

### Vaccine

The master seed bank and the working seed bank of the H7N9 vaccine virus were produced under good manufacturing practices (GMP) conditions. For the production of the split H7N9 vaccine antigen under GMP conditions, the diluted H7N9 vaccine strain from the working seed bank was inoculated into embryonated chicken eggs (10–11 days old) and incubated at 33–35 °C for 60 h. After incubation, the eggs were chilled at 2–8 °C and the allantoic fluid was manually harvested and clarified by centrifugation. The virions were purified by two consecutive ultracentrifugation steps of sucrose gradient, collected, diluted with phosphate buffer and fragmented by Triton X-100 treatment (0.5% final concentration). The split H7N9 was further clarified by centrifugation and diafiltration (cutoff 50 kDa, using phosphate buffer, 10 times the initial volume). After appropriate dilution, formaldehyde was added to 0.01% final concentration for inactivation. The H7N9 antigen was filtered in 0.22 µm filter and considered concentrated H7N9 vaccine antigen bulk. Quality control of H7N9 vaccine antigen was performed according to the WHO guidelines^67^ and the H7 haemagglutinin content of the bulk was determined by single radial immunodiffusion assay^67^ using reference anti-H7 hemagglutinin antibodies and H7 hemagglutinin antigen from the NIBSC (UK). The vaccines were prepared freshly from the bulk at the day of vaccination by diluting with phosphate-buffered saline (PBS) to the desired concentration. The adjuvanted vaccines were prepared by admixing the SWE adjuvant in a 1:1 volume ratio on site. All vaccines were for prepared in a volume of 0.5 mL.

### Adjuvant

SWE is a squalene-in-water emulsion adjuvant comprising a metabolizable oil (squalene 3.9%, w/v), sorbitan trioleate (0.47%, w/v), and polyoxyethylene (80) sorbitan monooleate (0.47%, w/v) dispersed in 10 mm citrate buffer at pH 6.5. Manufacturing of SWE was performed by adding an aqueous phase containing polyoxyethylene sorbitan monooleate in citrate buffer at pH 6.5 to the oil phase, containing squalene and sorbitan trioleate^35^. The mixture was homogenized with a high-shear mixer (Silverson L5M-A) at 8000 rpm for 2 min and immediately microfluidized (Microfluidics M-110EH) with five passes at 20,000 psi while cooling at 10 °C. The final emulsion was sterilized by filtration and characterized by assessing visual appearance, measuring pH, determining particle size by dynamic light scattering, squalene concentration by reversed-phase high-pressure liquid chromatography, endotoxin content, sterility, zeta potential, osmolarity, squalene oxidation, and presence of large particles.

### Ferrets and handling

Female ferrets (*Mustela putorius furo*), aged 6–12 months were confirmed to be negative for previous infection with circulating influenza virus and Aleutian disease. A temperature transponder (DST micro T, Star-Oddi) was implanted intra-abdominally 14 days prior to commencement of the experiment, which recorded the temperature every 30 min. For this purpose, sevoflurane was used as an anesthetic and buprenorphine (0.2 mL subcutaneously) as a post-operative analgesic. Blood sampling, immunizations, swabs, and challenge were performed under anesthesia by ketamine (5 mg/kg) and medetomidine hydrochloride (0.1 mg/kg) and the anesthesia was antagonized with atipamezole (0.25 mg/kg). After intratracheal challenge, ferrets were antagonized after 30 min to avoid sneezing and coughing reflexes. All animals had ad libitum access to pelleted standard ferret food and tap water.

### Immunization and challenge of ferrets

Groups were intended to include six ferrets each. However, two ferrets had to be terminated owing to a tumor development and a bacterial infection prior to start of the study and during the vaccination period, respectively. Ferrets were intramuscularly vaccinated (0.5 mL) twice at day 0 and 21 with a placebo, an H7N9 split vaccine at a dose of 15 or 45 μg HA or a SWE-adjuvanted H7N9 split vaccine at a dose of 7.5 or 15 μg HA. The 45 μg split vaccine group contained five ferrets from the start of the study and the 7.5 μg adjuvanted group contained five ferrets from Day 31 and onward. Serum samples were collected at regular time intervals (day −1, 13, 20, 37, and 41) and stored at −20 °C for serological analysis. Thirty-eight days after start of the study, the animals were challenged intratracheally with WT influenza A/Anhui/1/2013 (H7N9, 10^7^ TCID50 in 3 mL). This dose was obtained from a previously performed dose-titration infection experiment and resulted in the most consistent induction of lung pathology and disease. Prior to challenge and 2 and 3 days after challenge, throat swabs were taken and ferrets were weighed. Swabs were vortexed in 2 mL transport medium (15% sucrose, 2.5 μg/mL Fungizone, 100 U/mL Penicillin, 100 μg/mL Streptomycin, 250 μg/mL Gentamicin in PBS) and were stored at <−70 °C until further virological analysis. Ferrets were scored twice daily for the clinical parameters activity and impaired breathing according to the following scoring system: 0=active; 1=active when stimulated; 2=inactive and 3=lethargic and 0=normal breathing; 1=fast breathing and 2=heavy and stomach breathing, respectively.

At day 41, animals were killed by cardiac bleeding. During section, the trachea was clamped off after inhalation and the inflated lungs were isolated, weighed, examined for gross pathology and photographed. The middle section of the trachea (~1 cm) and a section of the three right lung lobes and the accessory lobe along the proximodistal axis (~1 cm by 3 mm) were isolated and stored at −70 °C for virological analysis. The left cranial and caudal lung lobes were fixed in 10% buffered formalin for histopathological analysis.

### Serological analysis

HAI titers in ferret sera were determined according to standard methods with a modification for H7N9 antibody responses^68^. Ferret sera were inactivated (30 min at 56 °C), treated with receptor destroying enzyme (RDE, Sigma-Aldrich), twofold serially diluted and tested in duplicate against four haemagglutinating units of the respective viruses using 1% horse red blood cells. The HI-titer was calculated by taking the average of the duplicate.

Virus neutralizing (VN) titers were determined as described elsewhere^69^. Sera were inactivated (30 min at 56 °C) and twofold serially diluted in virus growth medium (MEM medium (Gibco, 31095), 40 µg/ml gentamycin (Sigma), 0.01 m Tricin (Sigma) and 2 µg/ml TPCK-treated trypsin (Sigma)) using a starting dilution of 1:8. An equal volume of virus at a concentration of 100 TCID_50_ was added and the mixture was incubated for 2 h at 37 °C. In addition, a back titration of the virus stock used for incubation with the serum dilutions was prepared by ½log10 serial dilutions. Following, the virus-serum mixture and the back-titration samples were transferred to 96-well plates containing confluent Madin-Darby canine kidney (MDCK) cells and incubated for another 2 h at 37 °C, 5% CO_2_ after which the medium was refreshed. Plates were incubated until a back-titration plate reached cytopathic effects (CPE) at a titer of 100 TCID_50_ (4–5 days). The 50% virus neutralization titers per mL serum was calculated by the Reed and Muench method^70^. The neuraminidase inhibition (NI) assay was performed as described by Lambre et al.^71^. For this purpose, 96-well plates (Immulon 2HB, Thermo Scientific) were coated overnight with 100 μl of 50 μg/ml fetuin (Sigma-Aldrich, USA). Twofold serial dilutions of serum were prepared in duplicates and incubated with RN9/13-human A(H6N9) at a standardized neuraminidase activity (OD_450_ = 1 after 1 h incubation at 37 °C). Following, 100 μl of the virus/serum dilutions were transferred to the fetuin-coated wells. After 4 h incubation at 37 °C, the plates were washed with PBS/Tween20 (0.5%) and supplemented with 100 μl of peroxidase-labeled peanut lectin (2.5 μg/ml; Sigma-Aldrich, USA), incubated for 1 h at 37 °C and washed again. Peroxidase substrate (TMB, 100 μl) was added and the reaction was stopped after 10 min with 100 μl of 2 M sulfuric acid. The OD450 was read using the EL808, Bio-Tek Instruments Inc, USA and the NI-titer was determined from the last dilution with an OD450 ≤ (OD450 of the virus control − 3× SD).

### Animal temperature and weight

Temperature data were retrieved from the implanted temperature loggers with the Mercury (v4.91) software and consisted of measurements taken every 30 min. Body weight data consisted of a single measurement maximally once per day at the indicated days. Body weight and temperature baselines were calculated as the average body weight and temperature over a period of 2 (body weight) and 7 days (temperature) prior to infection or challenge. The change in temperature and body weight (ΔT and Δweight) was calculated by subtracting the baseline from the measurement after infection or challenge. The sum of all these measurement divided by the number of measurements is the average change in body weight or temperature. The maximum change in body weight or temperature was the maximum change compared to baseline recorded during any of the measurement after infection or challenge.

### Virus quantification

Lung and trachea samples were homogenized using FastPrep (MP Biomedicals) homogenizer and clarified by low speed centrifugation and transport medium from the swabs was used directly. Virus titers were determined by end-point titration on MDCK cells according to a protocol published by the WHO^69^. For this purpose, MDCK cells were seeded in 96-well plates at a density of 1–5 × 10^4^ cells/well and incubated at 37 °C until 90–100% confluence. Viral samples were five-fold serially diluted in 100 μL virus growth medium (MEM medium (Gibco, 31095), 40 µg/ml gentamycin (Sigma), 0.01 m Tricin (Sigma) and 2 µg/ml TPCK treated trypsin (Sigma)). The cells were inoculated in sextuplets with the titrated viral samples and after six days of incubation at 37 °C, wells were scored for CPE. The TCID_50_ titer was determined by the Reed and Muench^70^.

## Pathology

After fixation, the left cranial and caudal lung lobes were embedded in paraffin. Sections were cut at a thickness of 5 µm and stained with haematoxylin and eosin. Slides were examined microscopically with an objective of ×4 or ×10. For evaluation of the infection pathology, two pathology categories were defined that included parameters indicating damage of the epithelial lining and inflammation related parameters. These were analyzed for the different compartments of the lung: bronchi(oli), interstitium, alveoli, and blood vessels. Damage related parameters included hypertrophy, hyperplasia, flattened or pseudo squamous epithelia, necrosis and denudation of bronchi(oli) epithelium, hyperemia of septa and alveolar emphysema and hemorrhages. Inflammation related parameters included (peri)bronchi(oli)tis, interstitial infiltrate, alveolitis and (peri)vasculitis characterized by polymorphonuclear (PMN) cells, macrophages and lymphocytic infiltrate. These categories were semi-quantitatively scored on a scale of 0 (no aberrations) to 5 (severe damage) resulting in two scores: one for damage, and one for inflammation, each for the bronchi(oli), interstitium, alveoli, and blood vessels. At least 6 microscopic fields were scored for each lobe. The affected percentage lung surface was estimated with an objective of ×2. Finally, an end score was determined by taking into account the severity and percentage of tissue affected by the various parameters. Microscopic slides were randomized and scored blindly. Images were processed in Adobe Photoshop CC (v19.1.9) and only brightness and contrast was applied to the whole image.

### Phylogenetic tree

The phylogenetic tree was generated by the PhyML tool^72^ provided by the Influenza Research Database^73^.

### Statistical analysis

All statistical analyses were performed in R (v3.6.0)^74^. Virus titers and antibody titers were transformed by the logarithm to base 10 and the detection of differences between groups with respect to these parameters was carried out with the one-tailed Wilcoxon–Mann–Whitney (WMW) test provided in the coin package (v1.2.1)^75^. Differences with respect to the pathology scores were tested by an adapted version of the WMW test based on mid *p* values, which were validated for ordinal data with small sample sizes. Pairwise associations between various functional antibody responses and parameters of vaccine efficacy were tested by means of the permutation version of the ‘independence test’ implemented in the coin package^75^. For the correlation analysis, multiple testing corrections were carried out with the Benjamini–Hochberg method^76^ at a nominal false discovery rate of 5%. Curve fitting was performed using the DoseFinding package (v0.9.16)^77^. The logistic, sigmoid Emax, Emax, and linear models were tested and out of these the model with highest *R*^2^ was selected for fitting. Cluster tendency determination using the Hopkin's test, distance matrix computation by the Euclidian method and visualization was performed with the factoextra package (v1.0.5)^78^. Cluster analysis was performed using PAM included in the cluster package (v2.0.8)^79^ and cluster and silhouette plots were created using the factoextra package (v1.0.5)^78^.

### Reporting summary

Further information on experimental design is available in the Nature Research Reporting Summary linked to this article.

## Supplementary information

Supplementary Information

Reporting Summary

## Data Availability

The data that support the findings of this study are available from the corresponding author upon request.

## References

[1] Gao R (2013). Human infection with a novel avian-origin influenza A (H7N9) virus. N. Engl. J. Med..

[2] WHO. Influenza at the human-animal interface: Summary and assessment, 13 February to 9 April 2019. (2019).

[3] Gao HN (2013). Clinical findings in 111 cases of influenza A (H7N9) virus infection. N. Engl. J. Med..

[4] Li H (2018). Comparison of patients with avian influenza A (H7N9) and influenza A (H1N1) complicated by acute respiratory distress syndrome. Medicine.

[5] WHO. Human infection with avian influenza A(H7N9) virus – China: Update *Disease Outbreak News* (2018).

[6] Wang X (2017). Epidemiology of avian influenza A H7N9 virus in human beings across five epidemics in mainland China, 2013-17: an epidemiological study of laboratory-confirmed case series. Lancet Infect. Dis..

[7] Wang SJ, Liu XW, Shen X, Hua XG, Cui L (2018). Epidemiological and molecular analysis of avian influenza A(H7N9) virus in Shanghai, China, 2013-2017. Infect. Drug Resist..

[8] Zhou L (2017). Preliminary epidemiology of human infections with highly pathogenic avian influenza A(H7N9) Virus, China, 2017. Emerg. Infect. Dis..

[9] Teng Y (2018). Contact reductions from live poultry market closures limit the epidemic of human infections with H7N9 influenza. J. Infect..

[10] Shi J (2017). H7N9 virulent mutants detected in chickens in China pose an increased threat to humans. Cell Res..

[11] Health, F. A. P. A. H7N9 situation update. (2018).

[12] Chen J (2017). First genome report and analysis of chicken H7N9 influenza viruses with poly-basic amino acids insertion in the hemagglutinin cleavage site. Sci. Rep..

[13] Hou G (2018). Hemagglutinin characteristics, changes in pathogenicity, and antigenic variation of highly pathogenic H7N9 avian influenza viruses in China. J. Infect..

[14] Yamayoshi S (2018). Enhanced replication of highly pathogenic influenza A(H7N9) virus in humans. Emerg. Infect. Dis..

[15] Hatta M, Gao P, Halfmann P, Kawaoka Y (2001). Molecular basis for high virulence of Hong Kong H5N1 influenza A viruses. Science..

[16] Schrauwen EJ (2012). The multibasic cleavage site in H5N1 virus is critical for systemic spread along the olfactory and hematogenous routes in ferrets. J. Virol..

[17] de Vries RP (2017). Three mutations switch H7N9 influenza to human-type receptor specificity. PLoS Pathog..

[18] Xiang D (2018). Convergent Evolution of Human-Isolated H7N9 Avian Influenza A viruses. J. Infect. Dis..

[19] Kieny MP (2006). A global pandemic influenza vaccine action plan. Vaccine.

[20] WHO. Global pandemic influenza action plan to increase vaccine supply. WHO/IVB/06.13; WHO/CDS/EPR/GIP/2006.1 (2006).

[21] Tweed SA (2004). Human illness from avian influenza H7N3, British Columbia. Emerg. Infect. Dis..

[22] Fouchier RA (2004). Avian influenza A virus (H7N7) associated with human conjunctivitis and a fatal case of acute respiratory distress syndrome. Proc. Natl. Acad. Sci. USA.

[23] Belser JA (2007). Pathogenesis of avian influenza (H7) virus infection in mice and ferrets: enhanced virulence of Eurasian H7N7 viruses isolated from humans. J. Virol..

[24] Jackson LA (2015). Effect of varying doses of a monovalent h7n9 influenza vaccine with and without as03 and mf59 adjuvants on immune response: a randomized clinical trial. JAMA.

[25] Shen Y (2016). Safety, immunogenicity and cross-reactivity of a Northern hemisphere 2013-2014 seasonal trivalent inactivated split influenza virus vaccine, Anflu(R). Hum. Vaccines Immunother..

[26] Mulligan MJ (2014). Serological responses to an avian influenza A/H7N9 vaccine mixed at the point-of-use with MF59 adjuvant: a randomized clinical trial. JAMA.

[27] Wu UI (2017). Safety and immunogenicity of an inactivated cell culture-derived H7N9 influenza vaccine in healthy adults: A phase I/II, prospective, randomized, open-label trial. Vaccine.

[28] Reed SG, Orr MT, Fox CB (2013). Key roles of adjuvants in modern vaccines. Nat. Med..

[29] Del Giudice G, Rappuoli R, Didierlaurent AM (2018). Correlates of adjuvanticity: a review on adjuvants in licensed vaccines. Semin. Immunol..

[30] Madan A (2016). Immunogenicity and safety of an AS03-adjuvanted H7N9 pandemic influenza vaccine in a rndomized trial in healthy adults. J. Infect. Dis..

[31] Wong SS (2015). Impact of adjuvants on the immunogenicity and efficacy of split-virion H7N9 vaccine in ferrets. J. Infect. Dis..

[32] Chia MY (2015). Evaluation of MDCK cell-derived influenza H7N9 vaccine candidates in ferrets. PloS ONE.

[33] Hatta M (2018). Effectiveness of whole, inactivated, low pathogenicity influenza A(H7N9) vaccine against antigenically distinct, highly pathogenic H7N9 virus. Emerg. Infect. Dis..

[34] Collin N, Dubois PM (2011). The Vaccine Formulation Laboratory: a platform for access to adjuvants. Vaccine.

[35] Ventura R (2013). Technology transfer of an oil-in-water vaccine-adjuvant for strengthening pandemic influenza preparedness in Indonesia. Vaccine.

[36] Westdijk J (2013). Antigen sparing with adjuvanted inactivated polio vaccine based on Sabin strains. Vaccine.

[37] Castiglioni P (2017). Exacerbated leishmaniasis caused by a viral endosymbiont can be prevented by immunization with its viral capsid. PLoS Neg. Trop. Dis..

[38] Marcandalli J (2019). Induction of potent neutralizing antibody responses by a designed protein nanoparticle vaccine for respiratory syncytial virus. Cell.

[39] Younis SY (2018). Down selecting adjuvanted vaccine formulations: a comparative method for harmonized evaluation. BMC Immunol..

[40] Wang C (2018). A simian-adenovirus-vectored rabies vaccine suitable for thermostabilisation and clinical development for low-cost single-dose pre-exposure prophylaxis. PLoS Negl. Trop. Dis..

[41] WHO. Summary of status of development and availability of avian influenza A(H7N9) candidate vaccine viruses and potency testing reagents. (2019).

[42] Guo L (2014). Human antibody responses to avian influenza A(H7N9) virus, 2013. Emerg. Infect. Dis..

[43] Wong SS (2014). A single dose of whole inactivated H7N9 influenza vaccine confers protection from severe disease but not infection in ferrets. Vaccine.

[44] Krammer F (2014). Divergent H7 immunogens offer protection from H7N9 virus challenge. J. Virol..

[45] Belser JA, Eckert AM, Tumpey TM, Maines TR (2016). Complexities in ferret influenza virus pathogenesis and transmission models. Microbiol. Mol. Biol. Rev..

[46] Margine I, Krammer F (2014). Animal models for influenza viruses: implications for universal vaccine development. Pathogens.

[47] Bodewes R, Rimmelzwaan GF, Osterhaus AD (2010). Animal models for the preclinical evaluation of candidate influenza vaccines. Expert Rev. Vaccines.

[48] Kong H (2015). A live attenuated vaccine prevents replication and transmission of H7N9 virus in mammals. Sci. Rep..

[49] Chen Z (2014). Development of a high-yield live attenuated H7N9 influenza virus vaccine that provides protection against homologous and heterologous H7 wild-type viruses in ferrets. J. Virol..

[50] Zhu H (2013). Infectivity, transmission, and pathology of human-isolated H7N9 influenza virus in ferrets and pigs. Science.

[51] Belser JA (2013). Pathogenesis and transmission of avian influenza A (H7N9) virus in ferrets and mice. Nature.

[52] Watanabe T (2013). Characterization of H7N9 influenza A viruses isolated from humans. Nature.

[53] de Jonge J (2016). H7N9 live attenuated influenza vaccine is highly immunogenic, prevents virus replication, and protects against severe bronchopneumonia in ferrets. Mol. Ther..

[54] Feng Y (2015). Molecular pathology analyses of two fatal human infections of avian influenza A(H7N9) virus. J. Clin. Pathol..

[55] Huang JB (2015). Histopathological findings in a critically ill patient with avian influenza A (H7N9). J. Thorac. Dis..

[56] Yu L (2013). Clinical, virological, and histopathological manifestations of fatal human infections by avian influenza A(H7N9) virus. Clin. Infect. Dis..

[57] Kreijtz JH (2013). Low pathogenic avian influenza A(H7N9) virus causes high mortality in ferrets upon intratracheal challenge: a model to study intervention strategies. Vaccine.

[58] Kreijtz JH (2015). A single immunization with modified vaccinia virus Ankara-based influenza virus H7 vaccine affords protection in the influenza A(H7N9) pneumonia ferret model. J. Infect. Dis..

[59] Park, J. K. et al. Evaluation of preexisting anti-hemagglutinin stalk antibody as a correlate of protection in a healthy volunteer challenge with influenza A/H1N1pdm virus. *mBio* **9**10.1128/mBio.02284-17 (2018). 10.1128/mBio.02284-17 PMC5784259 29362240

[60] Sandbulte MR (2007). Cross-reactive neuraminidase antibodies afford partial protection against H5N1 in mice and are present in unexposed humans. PLoS Med..

[61] Rockman S (2013). Neuraminidase-inhibiting antibody is a correlate of cross-protection against lethal H5N1 influenza virus in ferrets immunized with seasonal influenza vaccine. J. Virol..

[62] Memoli MJ (2016). Evaluation of antihemagglutinin and antineuraminidase antibodies as correlates of protection in an influenza A/H1N1 virus healthy human challenge model. mBio.

[63] Maier HE (2019). Pre-existing anti-neuraminidase antibodies are associated with shortened duration of influenza A (H1N1)pdm virus shedding and illness in naturally infected adults. Clin. Infect. Dis..

[64] Gao, J. et al. Antigenic drift of the influenza A(H1N1)pdm09 virus neuraminidase results in reduced effectiveness of A/California/7/2009 (H1N1pdm09)-specific antibodies. *mBio* **10**, pii: e00307-19 (2019). 10.1128/mBio.00307-19 PMC6456748 30967460

[65] Rudenko L (2016). H7N9 live attenuated influenza vaccine in healthy adults: a randomised, double-blind, placebo-controlled, phase 1 trial. Lancet Infect. Dis..

[66] McLean KA, Goldin S, Nannei C, Sparrow E, Torelli G (2016). The 2015 global production capacity of seasonal and pandemic influenza vaccine. Vaccine.

[67] WHO. Recommendations for the production and control of influenza vaccine (inactivated). Report No. No. 927 (2005).

[68] WHO. Laboratory Procedures: Serological detection of avian influenza A(H7N9) virus infections by modified horse red blood cells haemagglutination-inhibition assay. (2013).

[69] WHO. WHO Manual on Animal Influenza Diagnosis and Surveillance; WHO/CDS/CSR/NCS/2002.5 Rev. 1. (2002).

[70] Reed LJ, H. Muench (1938). A simple method of estimating fifty per cent endpoints. Am. J. Hyg..

[71] Lambre CR, Terzidis H, Greffard A, Webster RG (1990). Measurement of anti-influenza neuraminidase antibody using a peroxidase-linked lectin and microtitre plates coated with natural substrates. J. Immunol. Methods.

[72] Guindon S, Gascuel O (2003). A simple, fast, and accurate algorithm to estimate large phylogenies by maximum likelihood. Syst. Biol..

[73] Zhang Y (2017). Influenza Research Database: an integrated bioinformatics resource for influenza virus research. Nucleic Acids Res..

[74] R Core Team. R: A language and environment for statistical computing. R Foundation for Statistical Computing, Vienna, Austria, http://www.R-project.org/ (2019).

[75] Torsten Hothorn KH, van de Wiel MA, Zeileis A (2008). Implementing a class of permutation tests: the coin package. J. Stat. Softw..

[76] Benjamini Y, Hochberg Y (1995). Controlling the false discovery rate: a practical and powerful approach to multiple testing. J. R. Stat. Soc. Ser. B.

[77] Bornkamp, B., Pinheiro, J. and Frank Bretz, F. DoseFinding: Planning and Analyzing Dose Finding Experiments v. R package version 0.9-16. https://CRAN.R-project.org/package=DoseFinding (2018).

[78] Kassambara, A., Mundt, F., Factoextra: Extract and Visualize the Results of Multivariate Data Analyses. https://CRAN.R-project.org/package=factoextra (2017).

[79] Maechler, M., Rousseeuw, P., Struyf, A., Hubert, M., Hornik, K. Cluster: Cluster Analysis Basics and Extensions. Rpackages. 1. (2019).

