# Supplementary material for: H7N9 influenza split vaccine with SWE oil-in-water adjuvant greatly enhances cross-reactive humoral immunity and protection against severe pneumonia in ferrets
Source: NPJ Vaccines. 2020 May 11;5:38. doi: 10.1038/s41541-020-0187-4 (PMC7214439; doi:10.1038/s41541-020-0187-4)
Supplement: Supplementary file 1 — Supplementary Information [file 41541_2020_187_MOESM1_ESM.pdf]

Supplemental figures and tables

H7N9 influenza split vaccine with SWE oil-in-water adjuvant greatly enhances cross-reactive humoral immunity and protection against severe pneumonia in ferrets

Jørgen de Jonge[1*], Harry van Dijken[1], Femke de Heij[1], Sanne Spijkers[1$], Justin Mouthaan[1#*], Rineke de Jong[2], Paul Roholl[3], Eduardo Alfredo Adami[4], Milena Apetito Akamatsu[5], Paulo Lee Ho[5], Livia Brunner[6] Nicolas Collin[6], Martin Friede[7] José A. Ferreira[8] and Willem Luytjes[1]

[1]Centre for Infectious Disease Control, National Institute for Public Health and the Environment (RIVM), Bilthoven, the Netherlands
[2]Central Veterinary Institute of Wageningen UR, Lelystad, the Netherlands
[3]Microscope Consultancy, Weesp, the Netherlands
[4] Influenza Vaccine Plant, Instituto Butantan, São Paulo, Brazil
[5]Seção de Vacinas Aeróbicas, Instituto Butantan, São Paulo, Brazil
[6]Vaccine Formulation Laboratory, University of Lausanne, Epalinges, Switzerland
[7]Initiative for Vaccine Research, World Health Organization, Switzerland.
[8]Department of Statistics, Informatics and Modelling, National Institute for Public Health and the Environment (RIVM), Bilthoven, The Netherlands.

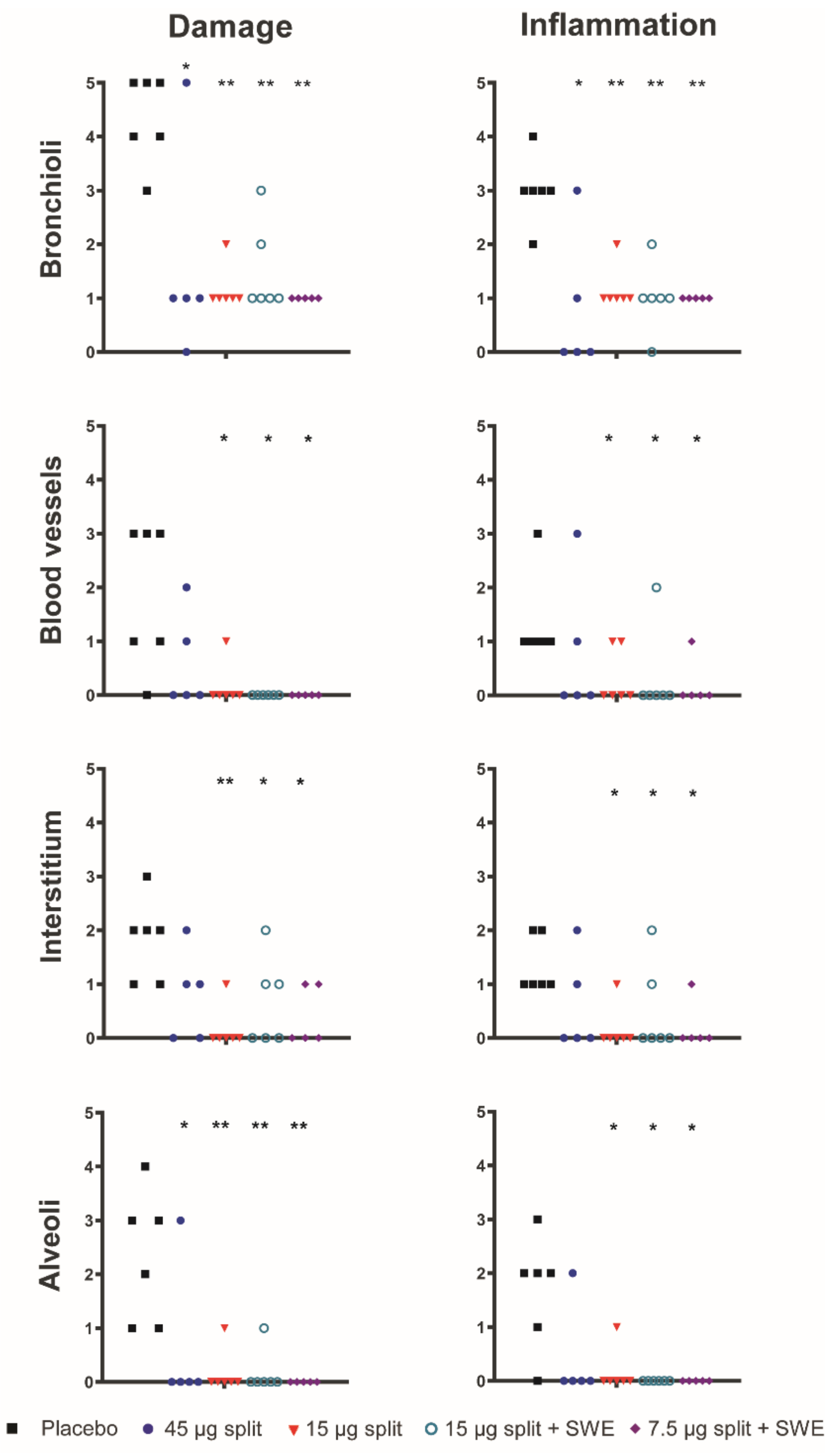

Supplemental figure S1. Detailed histopathological analysis of the lung.

Lung pathology was scored based on parameters that relate to damage (left graphs) and inflammation (right graphs) and these were assessed for the different lung compartments: bronchioli, blood vessels, interstitium and alveoli. The scores range from 0 (no aberrations) to 5 (severely affected). At least 6 random microscopic fields were analyzed and scores were averaged). Statistical analysis was performed using an adapted version of the WMW test: * p<0,05; **p<0,01 indicate difference compared to placebo.

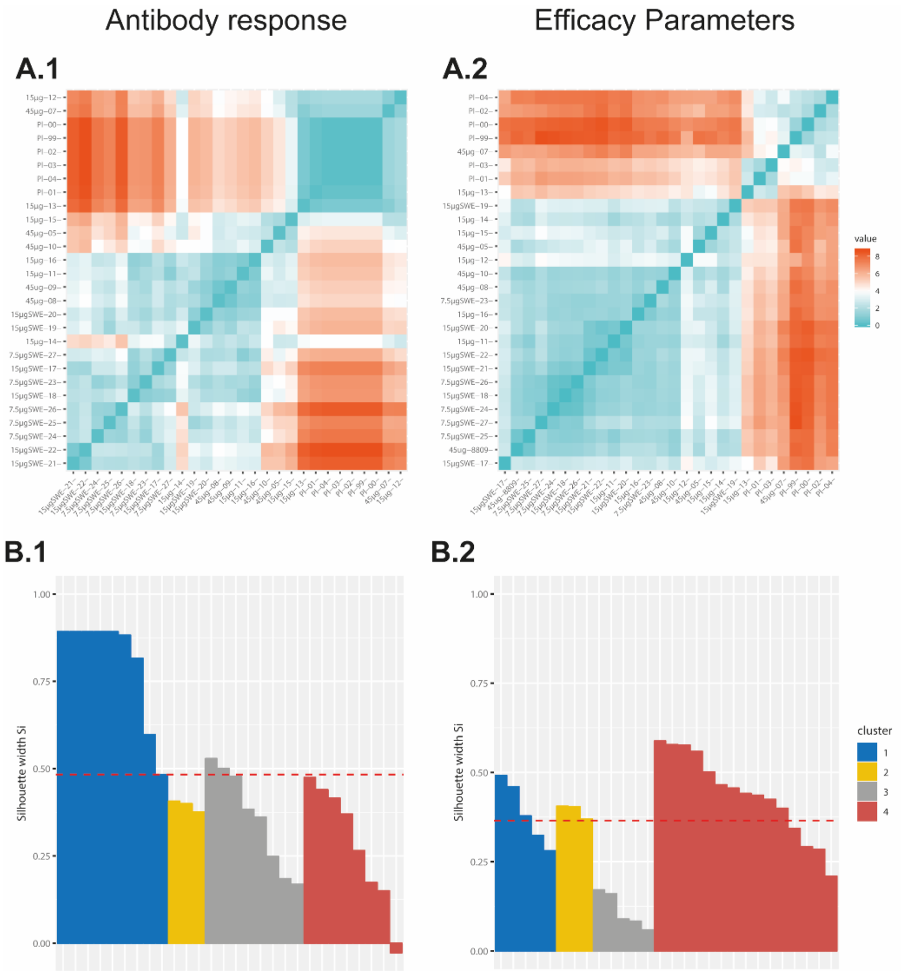

Supplemental figure S2. Determination of the number of clusters and validation of cluster analysis

Panel A represents dissimilarity matrices based on the Euclidean method in which the distance between two subjects is indicated by color; Blue indicates closely related and red indicate not related. When on the diagonal line, larger squares of similar color can be identified, this is an indication for a cluster. Distance in A.1 was calculated from the Area Under Curve (AUC) of the VN-, NI, and HI antibody log-titers from D0 until D37. Distance in A.2 was calculated from all the virus titration assays, weight and temperature measurements, and numerical pathology measurements (excluding ordinal, subjective data). Only numerical output data was used, since these are more reliable than ordinal scoring data and have statistically more power. In panel B a second validation is performed using a silhouette plot for the PAM cluster algorithm using 4 clusters. In this plot the likelihood of a samples belonging to a cluster is plotted. The higher the value, the more likely it is to belong to that cluster. If multiple subjects are below zero, this indicates that the number of clusters need reconsideration. Horizontal dashed line indicates the average silhouette width.

Supplemental Table S1. Clinical score and survival rates after challenge (day 39-41).

	**Activity**			**Breathing**			**Survival**	
	Total score/ group	# observations	Av score/ observation	Total score/group	# observations	Av score/ observation	# dead/total	#/d.p.c.
Placebo	19	21	0,90	18	21	0,86	3/6	1/2; 2/3
45µg Split	4	20	0,20	4	20	0,20	0/5	-
15µg Split	0	24	0,00	0	24	0,00	0/6	-
15µg Split+SWE	0	24	0,00	0	24	0,00	0/6	-
7,5µg Split+SWE	0	20	0,00	0	20	0,00	0/5	-

After challenge ferrets were scored twice daily for activity, impaired breathing and survival. The following scoring system was used for activity: 0=active; 1=active when stimulated; 2=inactive and 3=lethargic and for Respiratory distress: 0=normal breathing; 1=fast breathing and 2=heavy and stomach breathing.

Supplemental Table S2. Fever and weight loss after challenge with WT H7N9 ***(day 39-41).***

	**Average change during the challenge phase**		**Maximum change during the challenge phase**	
	ΔT (°C) [#]	Δweight (%) [#]	Max ΔT (°C) [#]	Max Δweight (%) [#]
Placebo	1.21 ± 0.21 [4]	-8.1 ± 3.62 [5]	3,07 ± 0.29 [5]	-10.3 ± 3.78 [5]
45µg Split	0.23 ± 0.19* [4]	-4.2 ± 5.8 [5]	1,42± 0.55**$ [6]	-4.6 ± 6.27* [5]
15µg Split	0.58 ± 0.70 [6]	-0.6± 2.78** [6]	2,25 ± 0.74* [6]	-0.0 ± 2.68** [6]
15µg Split+SWE	0.26** ± 0.18 [5]	-1.9 ± 2.67**## [6]	1,42**$$ ± 0.27 [5]	-1.3 ± 1.74**## [6]
7,5µg Split+SWE	-0.06**#$ ± 0.13 [5]	-0.6 ± 1.55** [5]	1,21**$± 0.49 [5]	-1.0 ± 1.41* [5]

[#] number of animals included for calculation; these vary between weight and temperature measurements since some transponders had a battery failure. Calculations excluded the ferret in the placebo group that died on day 40. The average baseline temperature per animal was calculated from day 33-37. The average ΔT was calculated by subtracting the baseline from the temperature measurements during the challenge period. The maximum increase was recorded from any of the measurement after challenge. Significant differences are indicated by * (compared to placebo), # (compared to 45µg split vaccines) and $ (compared to 15µg split vaccine). Differences were determined by the Mann-Whitney test: one symbol: p<0,05; two symbols: p<0,01.

Supplemental Table S3. Correlation analysis.

**VN-titer at Day 37**			
**variable**	**p.value**	**# of samples**	**FDR**
NI.D37	0	28	0
HI.D37	0	28	0
Virus.in.lungs	0	28	0
Affected.lungs.macro	0	28	0
Swabs.3dpc	1.00E-07	28	0
Virus.in.trachea	1.00E-07	28	0
RLW	3.00E-07	28	0
Alveolar.damage	6.80E-06	28	0
Damage.to.blood.vessels	7.50E-06	28	0
End.score	1.04E-05	28	0
Alveolar.inflammation	1.07E-05	28	0
Maximum.weight.loss	1.61E-05	27	0
Average.weight.loss	1.93E-05	27	0
Max.dT	9.14E-05	25	0
Bronchiolar.damage	9.40E-05	28	0
Swabs.2dpc	0.000132	28	0
Av.dT	0.000213	24	0
Affected.lungs.micro	0.000272	28	0
Inflammation.of.blood.vessels	0.000749	28	0.001
Damage.to.interstitium	0.002477	28	0.003
Inflammation.of.interstitium	0.015338	28	0.018
Inflammation.of.bronchi	0.015564	26	0.017
Bronchiolar.inflammation	0.030071	28	0.031
Damage.to.bronchi	0.185016	26	0.185

**NI-titer at Day 37**			
**variable**	**p.value**	**# of samples**	**FDR**
VN.D37	0	28	0
HI.D37	0	28	0
Swabs.3dpc	0	28	0
Virus.in.lungs	0	28	0
Virus.in.trachea	2.00E-07	28	0
Affected.lungs.macro	1.40E-06	28	0
RLW	4.32E-05	28	0
Damage.to.blood.vessels	8.77E-05	28	0
Alveolar.inflammation	0.000101	28	0
Alveolar.damage	0.000103	28	0
End.score	0.000112	28	0
Max.dT	0.000122	25	0
Swabs.2dpc	0.000199	28	0
Av.dT	0.000228	24	0
Maximum.weight.loss	0.000271	27	0
Bronchiolar.damage	0.000363	28	0.001
Affected.lungs.micro	0.000423	28	0.001
Average.weight.loss	0.000515	27	0.001
Inflammation.of.blood.vessels	0.006446	28	0.008
Damage.to.interstitium	0.01219	28	0.015
Inflammation.of.interstitium	0.03663	28	0.042
Inflammation.of.bronchi	0.058412	26	0.064
Bronchiolar.inflammation	0.178161	28	0.186
Damage.to.bronchi	0.326621	26	0.327

HI-titer at Day 37			
**variable**	**p.value**	**# of samples**	**FDR**
NI.D37	0	28	0
VN.D37	0	28	0
Swabs.3dpc	2.80E-06	28	0
Virus.in.lungs	4.73E-05	28	0
Virus.in.trachea	7.47E-05	28	0
Max.dT	9.90E-05	25	0
Swabs.2dpc	0.000126	28	0
Maximum.weight.loss	0.000343	27	0.001
Average.weight.loss	0.000391	27	0.001
Affected.lungs.macro	0.000694	28	0.002
Av.dT	0.000812	24	0.002
RLW	0.001184	28	0.002
End.score	0.00143	28	0.003
Damage.to.blood.vessels	0.001581	28	0.003
Alveolar.inflammation	0.001787	28	0.003
Alveolar.damage	0.00214	28	0.003
Bronchiolar.damage	0.007929	28	0.011
Affected.lungs.micro	0.009836	28	0.013
Inflammation.of.blood.vessels	0.015815	28	0.02
Damage.to.interstitium	0.034789	28	0.042
Inflammation.of.bronchi	0.052297	26	0.06
Inflammation.of.interstitium	0.122473	28	0.134
Bronchiolar.inflammation	0.194619	28	0.203
Damage.to.bronchi	0.338409	26	0.338

P-values of the tests of independence between functional antibody titers two weeks after booster (Day 37) and each of various response parameters. Using the Benjamini- Hochberg test for multiple testing at a nominal false discovery rate (FDR) of about 5% we conclude that the top 23, 22 and 21 associations with VN-, NI and HI-titers respectively are genuine except perhaps for three or four ((23+22+21)×0.05=3.3).
